# Visualization of the interaction of water aerosol and nanofiber mesh

**DOI:** 10.1063/5.0061847

**Published:** 2021-09-07

**Authors:** Boyang Yu, Jian Chen, Daner Chen, Rouxi Chen, Yuenan Wang, Xiujuan Tang, Hsing-Lin Wang, Lian-Ping Wang, Weiwei Deng

**Affiliations:** 1 Department of Mechanics and Aerospace Engineering, Southern University of Science and Technology, Shenzhen 518055, China; 2 Department of Materials Science and Engineering, Southern University of Science and Technology, Shenzhen 518055, China; 3 Peking University Shenzhen Hospital, No. 1120, Lianhua Road, Shenzhen 518036, China; 4 Shenzhen Center for Disease Control and Prevention, Shenzhen 518055, China

## Abstract

Face masks play a critical role in reducing the transmission risk of COVID-19 and other respiratory diseases. Masks made with nanofibers have drawn increasingly more attention because of their higher filtration efficiency, better comfort, and lower pressure drop. However, the interactions and consequences of the nanofibers and microwater droplets remain unclear. In this work, the evolution of fibers made of polymers with different contact angles, diameters, and mesh sizes under water aerosol exposure is systematically visualized. The images show that capillarity is very strong compared with the elasticity of the nanofiber. The nanofibers coalesce irreversibly during the droplet capture stage as well as the subsequent liquid evaporation stage. The fiber coalescence significantly reduces the effective fiber length for capturing aerosols. The nanofiber mesh that undergoes multiple droplet capture/evaporation cycles exhibits a fiber coalescing fraction of 40%–58%. The hydrophobic and orthogonally woven fibers can reduce the capillary forces and decrease the fiber coalescing fraction. This finding is expected to assist the proper design, fabrication, and use of face masks with nanofibers. It also provides direct visual evidence on the necessity to replace face masks frequently, especially in cold environments.

## I. INTRODUCTION

The COVID-19 pandemic has infected over 180 million and claimed the lives of nearly 4 million people (as of June 2021) since its outbreak in 2019.^1^ The consensus has been reached by the scientist community^2^ as well as the World Health Organization^1^ and the Centers for Disease Control and Prevention (CDC) in the United States^3^ that aerosol could be a route for the COVID-19 virus transmission. Aerosol refers to the droplets and droplet nuclei that are smaller than a certain size (say 5–10 *μ*m in diameter, for example). The COVID-19 infected patient generates a significant amount of aerosol via breathing activities such as coughing, sneezing, talking, singing, and breathing.^4,5^ Despite the massive campaigns of vaccination in many countries, the face masks are still the simplest and most accessible way of personal protection.

The majority of the filtration layers of face masks are made through the melt blown process with polypropylene (PP). The typical melt blown PP fiber diameter is 3–10 *μ*m. Recently, the nanofiber has emerged as a high-performance filtration material.^6,7^ The nanofibers are usually made via electrospinning from polymer solutions. The diameter of the nanofibers ranges from a few tens of nanometers to sub-micrometer. The small fiber diameter renders unique and favorable characteristics of the nanofiber based filtration materials, including: (i) the low flow resistance and low pressure drop due to possible slip effects^8,9^ as the diameter of the fiber is comparable with the mean free path of the air (∼100 nm at 1 atm); (ii) the light weight due to a high surface area to mass ratio; (iii) the semi-transparency owing to small fiber diameters that are less than the visible light wavelength; and (iv) the strong built-in electrostatic field *E_s_* near the fiber surface that further enhances the aerosol capturing capability, as *E_s_* is inversely proportional to the fiber radius.

The basic process of blocking aerosols is the capturing of the small droplets by the nanofibers.^10^ Droplets around the face mask may come from at least three sources. The first is the violent breathing activities such as coughing and talking. The second is the condensation of warm exhaled water vapor in cold air during normal breathing. The exhaled gas is nearly saturated with water vapor at the body temperature of 37 °C. Upon meeting the cold air, the warm and saturated water vapor condensates into numerous water droplets instantaneously and spontaneously. Nanofibers may encounter these droplets during each breathing cycle in cold environments. The third source may come from sterilization practices. When the medical supply is scarce, the need for re-use of masks leads to many protocols for sterilizing used masks^11^ such as boiling, steaming, and spraying. These sterilizing practices may generate droplets to be captured by the fibers.

One potential concern for both normal wearing and sterilization processes is whether the mechanical strength of the nanofiber is sufficiently strong to withstand the capillarity by the captured droplets. This is a legitimate concern, because fibers wetted by droplets tend to deform due to the elasticity combined with high aspect ratios under capillary forces. The competition between elasticity and capillarity has been studied by several groups. Princen^12^ investigated the cohesive force between two parallel fibers for different shapes of liquid bridges. Bedarkar *et al.*^13^ numerically investigated the effect of contact angle on the length of the droplet. Aziz and Tafreshi^14^ studied the cohesive force of both parallel and crossed fibers. Sauret *et al.*^15^ studied the effect of cross angle on the wetted length of two crossed fibers. The work by Stone and co-workers^16^ has shown that flexibility and drop size are the crucial parameters. They found that there is a critical drop volume, above which this coalescence does not occur. It is reasonable to expect that nanofibers are prone to coalesce, because the aerosol droplets are small and the nanofiber is extremely flexible. However, despite the rich literature on the microfiber wetting^12–23^ and effectiveness test of masks,^11,24–31^ very few papers have shown visually the interaction between aerosol droplets and nanofibers. This work is motivated by the need for visually observing the interaction between the nanofiber and water aerosols. We aim to obtain direct experimental evidence on the behavior of the nanofibers under the flow of aerosol with droplets of a few micrometers in diameter. We show that while aerosol droplets are effectively captured by fresh nanofibers, the capillarity leads to irreversible coalescence of fibers. The coalescence of fibers is more severe during the drying stage, as the liquid bead shrinks into a liquid bridge. Among different materials tested, nanofibers made of materials of high hydrophobicity are less prone to coalesce due to higher contact angles and consequently weaker capillary forces. In addition, the orthogonally woven fibers are more resilient to capillarity. This work may offer guidelines for making more robust face masks of nanofibers and also provides direct visual evidence on the necessity to replace face masks frequently, especially in cold environments.

## II. EXPERIMENTAL

**FIG. 1.**
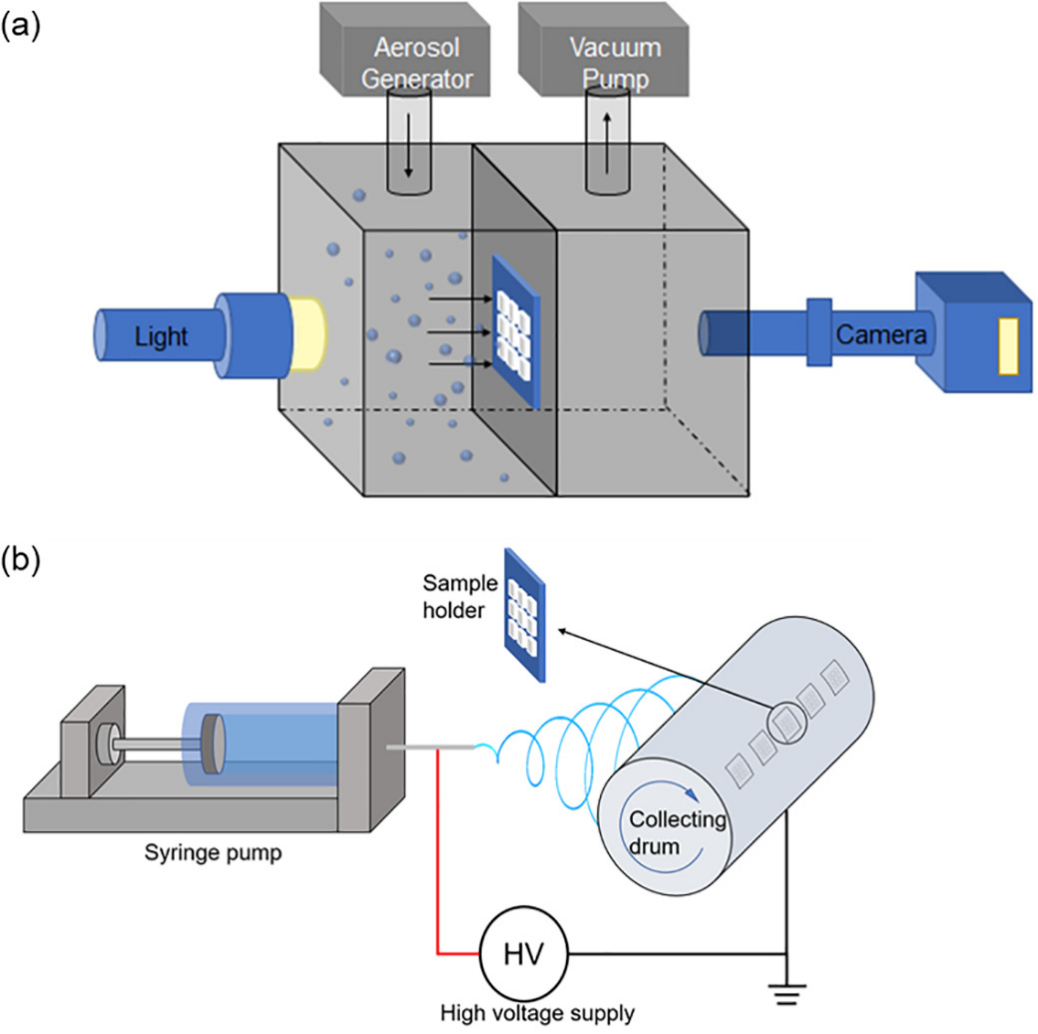
The experimental setup for (a) visualizing the interaction of water droplets and nanofiber mesh and (b) preparing the nanofiber samples via electrospinning.

The experimental setup [Fig. 1(a)] consists of the aerosol generator, test chamber, high-speed camera, and light source. The test chamber has a notch to accommodate the sample holder for the nanofibers. The sample holder is a square aluminum plate (15 × 15 × 0.1 mm^3^) with a 3 × 3 array of square openings etched on it by a laser marker. Each opening is measured at 500 × 500 *μ*m^2^. The fibers are spun directly onto the sample holder fixed on a rotating collection drum of an electrospinning apparatus [Fig. 1(b)]. Three types of polymers with different hydrophobicity are tested: polyvinyl alcohol (PVA), polyacrylonitrile (PAN), and polyvinylidene fluoride (PVDF). The high voltage applied is 10–20 kV, and the relative humidity (RH) is 50%–60%. The fiber diameter can be tuned by adjusting the polymer solution flow rate and polymer concentration. Typical polymer concentration is 6%–10% (PVA), 10%–14% (PAN), and 21%–25% (PVDF). The typical flow rate is: 0.1–0.5 ml/h (PVA) and 0.5–1.0 ml/h (PAN and PVDF). The produced fiber diameter ranges from ∼60 nm to ∼1 *μ*m. The fiber number density can be roughly adjusted by changing the collection time. The fiber alignment can be improved (i.e., more parallel) by increasing rotating speeds of the drum. We can also change the sample holder's fixing orientation on the drum to obtain approximately orthogonal fibers.

In the test chamber, a middle partition divides the chamber into two parts. The left chamber is filled with tap water aerosol produced by an ultrasonic atomizer, and the averaged diameter is 5 *μ*m. A carrier gas with a flow rate of 5 l/min is used to dilute and carry the aerosol to pass the sample holder. After ∼10 s, the ultrasonic atomizer is shut off while the carrier gas remains on for another ∼10 s. This cycle may be repeated for multiple times. To visualize the interaction of the aerosol and nanofibers, we used a high-speed camera (IX-Camera iSpeed 220) coupled with a long working distance microscopic object lens (Mitutoyo M Plan Apo SL 20×). The working distance of the lens is 30.5 mm; the depth-of-field is about 3 *μ*m; and the resolving power is 0.7 *μ*m. The imaging system has a field of view of 300 × 300 *μ*m^2^. The frame rate of the high-speed camera is 4000 frames per second at the resolution of 608 × 600. An LED light source projects a collimated white illumination from the back to form the shadowgraph configuration.

## III. RESULTS AND DISCUSSION

### A. Nanofiber coalescence and coalescing fraction

**FIG. 2.**
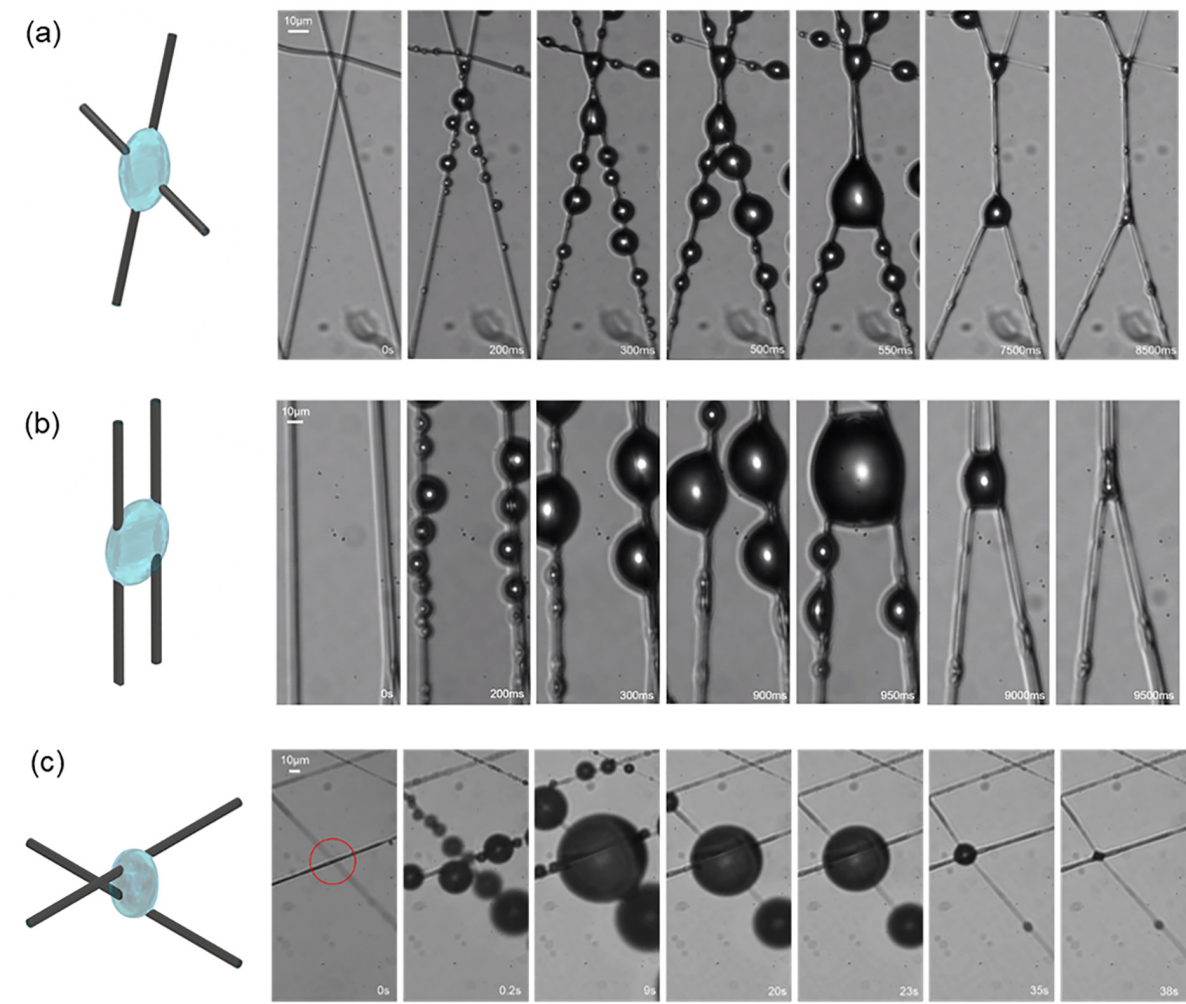
Three typical configurations of fiber arrangements and their response to the aerosol. (a) Crossed fibers (PVA). (b) Parallel or nearly parallel fibers (PVA). (c) Unparallel and untouched fibers (PVDF). All three configurations undergo two main stages: aerosol capture and liquid evaporation. Scale bar: 10 *μ*m.

Figure 2 shows the typical interactions of the aerosol and nanofibers for three basic fiber configurations:

(i) Crossed: Two straight fibers touch each other at an intersection point with an angle *θ*. This is the most common configuration of two fibers. The crossed fibers make up the majority of the mesh sample and eventually the filter mat used in face masks.
(ii) Parallel or nearly parallel: In this configuration, two fibers are on the same plane, and they are nearly parallel to each other without touching (at least in the field of view).
(iii) Unparallel and untouched: Two fibers are spatially separated and not parallel. For example, in Fig. 2(c), only one fiber is in the focal plane, while the other fiber is out of focus. Because the depth-of-field of the object lens is ∼3 *μ*m, the two unparallel fibers are at least a few micrometers apart.

The response of the nanofibers to the aerosol of all these three configurations involves two main stages: the first stage is aerosol capture, followed by the second stage of liquid evaporation. For the crossed fibers [Fig. 2(a)], the aerosol droplets are first captured by each fiber to form strings of liquid beads. These beads grow gradually, and then the beads from two fibers touch and merge, pulling the two fibers together. During the drying stage, the shrinking beads become a spreading meniscus that brings both fibers closer and eventually bind them together. The start of fiber binding is marked by the transition from barrel-shape (both fibers' circumferences are entirely emerged inside the droplet) to bridge (only part of the fibers' perimeter is wetted by the liquid).^16^

For the nearly parallel fibers [Fig. 2(b)], the small droplets that makeup the aerosol are captured by the fiber and form strings of microbeads. The neighboring beads on the same fiber will merge to form larger ones. When the bead radius grows till it touches the other fiber, the liquid bead becomes a liquid barrel connecting these two fibers, and the capillary force pulls both fibers toward each other. During the liquid evaporation stage, the liquid barrel shrinks into a liquid bridge and exerts stronger capillary force that bonds the two fibers together.

For the unparallel and untouched fibers [Fig. 2(c)], the process is similar to the parallel fibers: the droplets first deposit on the fiber and form growing beads and then the liquid beads eventually connect two fibers. During the evaporation stage, the liquid bridge shrinks and keeps the fibers tightly bonded [as both fibers come to the focal plane as shown in the last image in Fig. 2(c)].

We note that for all three fiber configurations, the coalescence of fibers is irreversible. That is, even after the liquid is fully evaporated, the fibers remain bond together, possibly due to the van der Waals force. Such irreversible fiber coalescence has strong implication on the filtration performance, which is directly related to the total length of the fibers for a given area, because when two fibers are fused together, the ability for capturing aerosol droplets decreases accordingly. Therefore, the coalescence of fibers deteriorates the filtration performance of the nanofiber mesh. To quantify the degree of coalescence, we define the coalescing fraction *η* by comparing the total fiber length reduction before and after the interaction of the aerosol with the fibers

$$\eta =1-\frac{L_{a}}{L_{0}}.$$ (1)

Here, *L*_0_ is the total length of the original dry fibers, and *L_a_* is the total length of the fibers after the capturing of aerosol and subsequent evaporation. Experimentally, we first calibrated the image to obtain the actual length represented by each pixel, and then we use the software ImageJ^32^ to count the length (first in terms of number of pixels that are then converted into actual length using the calibration data) of each fiber. Summing up all fiber lengths gives the total fiber length.

### B. The effect of contact angle and diameter on fiber coalescence

**FIG. 3.**
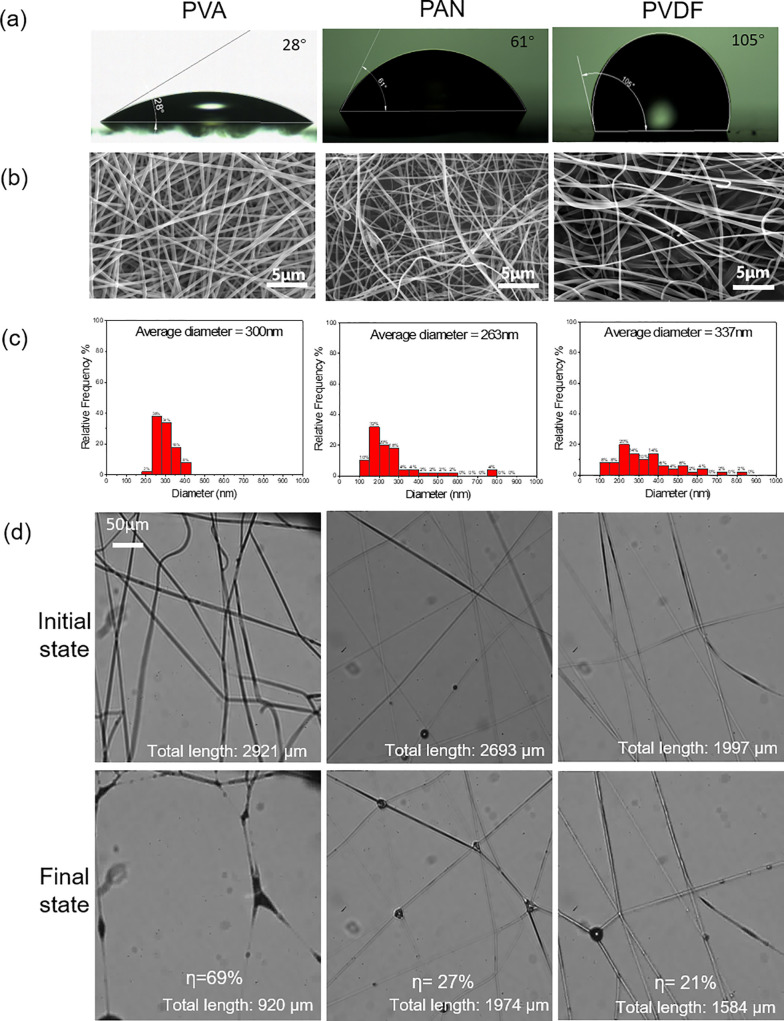
The effect of fiber hydrophobicity on the fiber coalescence. (a) Contact angle measurement for three fibers; (b) SEM images of the fibers; (c) the fiber diameter histogram; and (d) optical images of the fresh fibers and the fibers that experienced a complete cycle of aerosol capture and drying.

Nanofibers can be made by numerous polymers via electrospinning. Those fibers vary in hydrophobicity as well as diameter. We first quantify the degree of hydrophobicity of three common polymers (PVA, PAN, and PVDF) by measuring the contact angle of the smooth films by spin coating at 3000 rpm. The measured contact angles are 28°, 61°, and 105° for PVA, PAN, and PVDF, respectively [Fig. 3(a)]. Then we make nanofiber mesh samples with comparable fiber diameter. It is worth mentioning that although the nanofibers are optically visible in our images, their diameter is below the diffraction limits of the imaging lens; therefore, the optical image cannot provide accurate fiber diameter information. Detailed fiber diameter is obtained via post processing of the scanning electron microscope (SEM) images [Fig. 3(b)] with ImageJ.^32^ We managed to make three types of fibers of comparable diameters (300, 263, and 337 nm). Next, we observe the fiber interactions with the aerosol. After each sample undergoes a complete cycle of aerosol capture and liquid evaporation, we measure the total fiber length and evaluate the coalescing fraction using Eq. (1). The coalescing fraction of PVA is very high at 69%, while for PAN the coalescing fraction is decreased to 27% (average *η* for four samples is 52%). With the PVDF fiber, the coalescing fraction is further reduced to 21% (average *η* for four samples is 29%). Clearly, the fiber hydrophobicity strongly affects the fiber coalescence. More hydrophilic fibers are more prone to capillarity-induced coalescence. This suggests that the hydrophilic nanofiber is not well suited for making face masks as the water droplets cause severe fiber coalescence and mesh coarsening that significantly reduce the filtration performance.

We also found that the coalescing fraction is insensitive to the fiber diameter, especially for hydrophilic materials. The fiber diameter can be enlarged by increasing the concentration of the polymer solution. We made PVA fibers with three different fiber diameters: 255, 631, and 1575 nm. We found that even with the fiber diameter increased by six times, there are still severe coalescence of fibers and dramatic reduction in effective fiber length. This is somewhat surprising, because the second moment of inertia *I*, which describes the bending rigidity of the fiber, scales with the fourth power of fiber radius (*I* ∝ *r*^4^) The increase in six times in diameter will increase the moment by three orders of magnitude (6^4^–10^3^). To better understand this finding, we estimate capillary-bending length *l_b_*, which is a characteristic length to assess whether a rod will deform under capillarity^33^

$$l_{b}=\;{\left(EI/p\gamma \right)}^{1/2},$$ (2)

where *E* is the Young modulus of the material, *I* is the second moment of inertia, *p* is the perimeter of the cross section of the fiber, and *γ* is the liquid surface tension. For a cylinder with radius *r*,

$$l_{b}=\;{\left(Er^{3}/8\gamma \right)}^{1/2}.$$ (3)

**FIG. 4.**
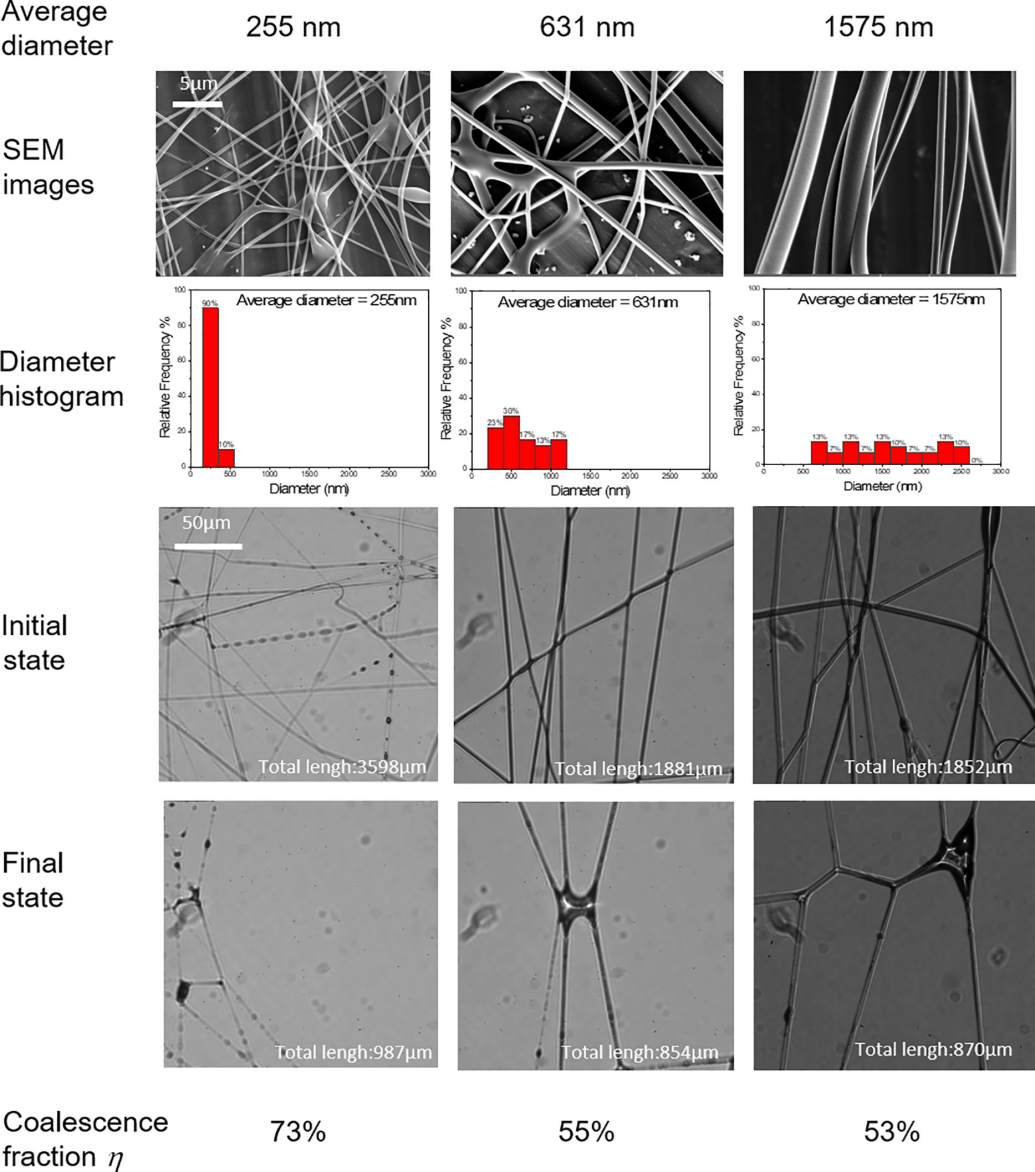
The effect of fiber diameter on coalescing fraction (PVA).

Equation (3) suggests that *l_b_* scales with *r*^3/2^, which is a much weaker dependence on *r* than that of second moment of inertia. For the polymer fiber, *E* ∼ 1 GPa and *l_b_* ∼ 2 *μ*m for 2*r* = 255 nm. If the fiber diameter increased to 2*r* = 1575 nm, then the capillary-bending length is increased to *l_b_* ∼ 30 *μ*m. Fibers that are much longer than the elastocapillary length will deform. The fiber length in Fig. 4 is ∼100 *μ*m > *l_b_*, which suggests the fibers tend to deform and helps us to explain the insensitivity of the coalescence fraction on the fiber diameter. The experimental data and the analysis suggest that for hydrophilic fibers, simply increasing the fiber diameter only has modest improvement in reducing the coalescing fraction.

### C. The effect of cross angle

**FIG. 5.**
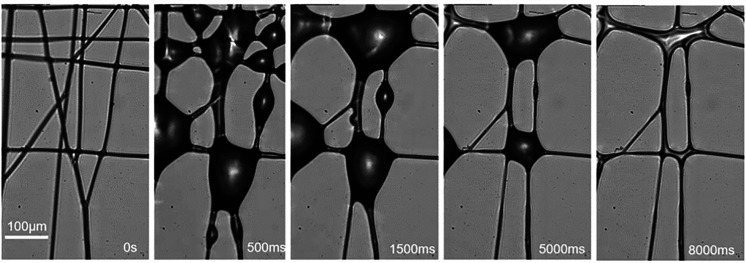
The wetted PVA fibers tend to form orthogonal bundles.

We also examined the effect of cross angle *θ* between fibers. Figure 5 shows one sample of multiple fibers that form several cross angles between each pair of fibers. After one complete cycle of aerosol capture and liquid evaporation, fibers with small angles coalesce, while the nearly orthogonal fibers survive.

We can understand the phenomena in Fig. 5 by the following argument. For parallel or crossed fibers, the cohesive force *F* scales with the wetted length *L_w_*. We first assume a liquid bridge forms between fibers. If the original liquid bead volume is *V*, we can estimate the maximum possibly wetted length *L_w_* by assuming that the liquid bridge formed between crossed fibers has the same surface area as the original liquid bead: *L_w_*^2^ sin*θ* ∝ *V*^2/3^, hence

$$L_{w}\propto \frac{V^{\frac{1}{3}}}{\;\text{sin}\;\theta^{\frac{1}{2}}}.$$ (4)

Therefore, it is clear that the cross angle dictates the magnitude of cohesive force, and the force reaches the minimum when the fibers are orthogonal. On the other hand, Wu *et al.*^34^ investigated the evolution of the minimum of the surface energy *E* as a function of the tilting angle. They found that the surface energy is lower in the web form for small tilting angle (<10°), and for larger tilting angle, a drop is likely to form. For a liquid drop instead of a bridge, the wetting length *L_w_* ∝ *V*^1/3^, which is the special case of relationship (4). Our observation is also consistent with the work by Aziz and Tafreshi,^14^ who found that the cohesive force of orthogonal fibers is weaker than that of parallel fibers in their experiment. For example, they found that the force of orthogonal fibers (*θ* = 90°) is only about 1/3 of the parallel fibers (*θ* = 0°). Therefore, orthogonal fibers lead to weakest cohesive force, and consequently, the right-angle intersection tends to be intact after the interaction with aerosol.

### D. Multiple cycles of wetting/drying

**FIG. 6.**
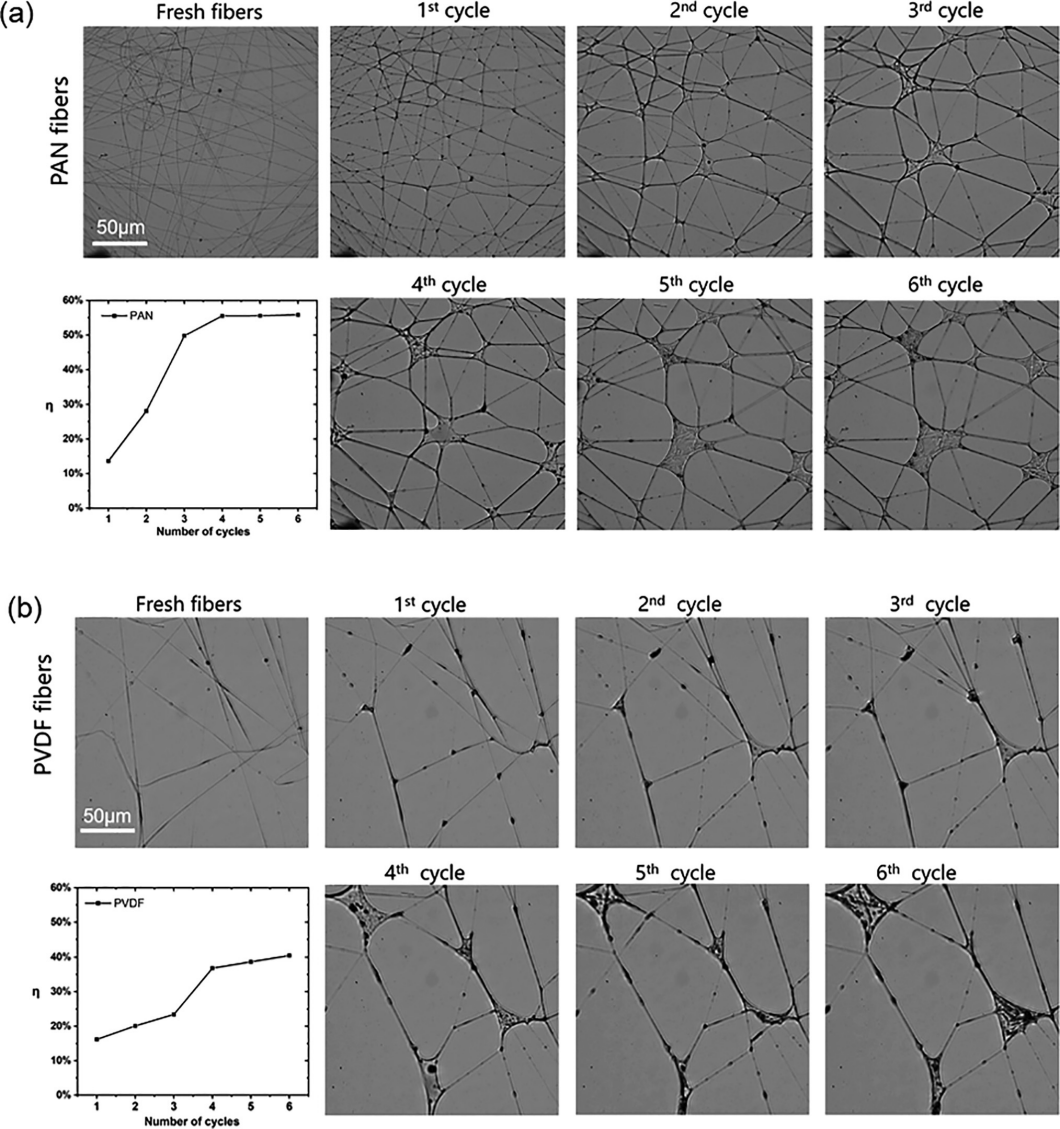
The change in mesh morphology and coalescing rate of (a) PAN fibers and (b) PVDF fibers after multiple cycles of aerosol capture and drying.

In an actual face mask, the filtering materials are likely to experience multiple cycles of aerosol capture and evaporation. To gain an understanding of how fibers respond to multiple such cycles, we investigated nanofiber meshes made by two materials (PAN and PVDF). Figure 6 shows the change in mesh morphology and coalescing fraction of PAN and PVDF nanofibers after multiple cycles of aerosol capture and evaporation.

For a PAN nanofiber mesh shown in Fig. 6(a), each of the first three cycles causes about 15%–20% increase in the coalescing fraction. The increase in *η* plateaus at about 58%. In other words, after multiple wet-dry cycles, about 2/3 total fiber length is lost due to capillarity induced fiber coalescence. For a PVDF nanofiber mesh shown in Fig. 6(b), the coalescing fraction increase per cycle is less than that of the PAN, and *η* stabilizes at about 40%. The result of multiple cycle test is consistent with the single cycle test of materials of different hydrophobicity: the more hydrophilic materials are more prone to coalesce. Eventually, the fiber arrangement evolves into a stabilized pattern that appears to be robust with respect to capillary cohesive force.

## IV. CONCLUSION

We investigated the consequence of nanofibers made of polymers of different hydrophobicity, diameters, and different mesh sizes under water aerosol exposures and subsequent drying. The aerosol droplets are effectively captured by fresh nanofibers, and the capillarity quickly leads to the coalescence of fibers. The fiber coalescence is more severe during the drying stage as the liquid bead shrinks to form a liquid bridge. Thicker fibers can withstand capillary forces slightly better than finer nanofibers. The fiber coalescence appears to be irreversible. Nanofibers made of materials of high hydrophobicity are less prone to coalesce due to higher contact angles and consequently weaker capillary force. In addition, the orthogonally woven fibers are more resilient to capillarity and less likely to coalesce. Nanofiber meshes that experience multiple cycles of aerosol capture and evaporation can lose about 40%–58% of the total fiber length due to fiber coalescence. This work provides direct visual evidence on the necessity to replace masks frequently, especially in cold environments where water vapor condensations tend to occur. This work also offers general guidelines for making more robust masks of nanofibers, that is, to use hydrophobic materials to make the nanofibers and form orthogonally woven meshes.

## AUTHORS' CONTRIBUTIONS

B.Y. and J.C. contributed equally to this work.

## Data Availability

The data that support the findings of this study are available within the article.
